# Robust topological designs for extreme metamaterial micro-structures

**DOI:** 10.1038/s41598-021-94520-x

**Published:** 2021-07-27

**Authors:** Tanmoy Chatterjee, Souvik Chakraborty, Somdatta Goswami, Sondipon Adhikari, Michael I. Friswell

**Affiliations:** 1 College of Engineering, Swansea University, Bay Campus, Swansea, SA1 8EN UK; 2 Department of Applied Mechanics, Indian Institute of Technology Delhi, Hauz Khas, 110016 India; 3 Department of Applied Mathematics, Brown University, Providence, RI 02912 USA

**Keywords:** Engineering, Materials science

## Abstract

We demonstrate that the consideration of material uncertainty can dramatically impact the optimal topological micro-structural configuration of mechanical metamaterials. The robust optimization problem is formulated in such a way that it facilitates the emergence of extreme mechanical properties of metamaterials. The algorithm is based on the bi-directional evolutionary topology optimization and energy-based homogenization approach. To simulate additive manufacturing uncertainty, combinations of spatial variation of the elastic modulus and/or, parametric variation of the Poisson’s ratio at the unit cell level are considered. Computationally parallel Monte Carlo simulations are performed to quantify the effect of input material uncertainty to the mechanical properties of interest. Results are shown for four configurations of extreme mechanical properties: (1) maximum bulk modulus (2) maximum shear modulus (3) minimum negative Poisson’s ratio (auxetic metamaterial) and (4) maximum equivalent elastic modulus. The study illustrates the importance of considering uncertainty for topology optimization of metamaterials with extreme mechanical performance. The results reveal that robust design leads to improvement in terms of (1) optimal mean performance (2) least sensitive design, and (3) elastic properties of the metamaterials compared to the corresponding deterministic design. Many interesting topological patterns have been obtained for guiding the extreme material robust design.

## Introduction

Metastructures are metamaterial induced concepts implanted in structural design. The exploration of metamaterials was heavily steered by the path-breaking investigation of electromagnetic metamaterials, which exhibited properties like, negative permittivity and permeability^1^. This paved the way for mechanical metamaterials^2^. These metamaterials are engineered to derive their fundamental mechanical properties from the geometry of their structural building blocks (periodicity or translational symmetry), instead of the constituting materials^3,4^. Mechanical metamaterials have gained wide popularity in engineering applications due to their superior properties compared to conventional materials found in nature. It has been found that by varying one or more material constants such as, bulk modulus, shear modulus, Poisson’s ratio and elastic modulus, extraordinary materials such as, ultra light weight and high strength aerospace shells, functionally graded electromagnetic sensors and energy absorbing dampers can be secured^5,6^.

**Figure 1:**
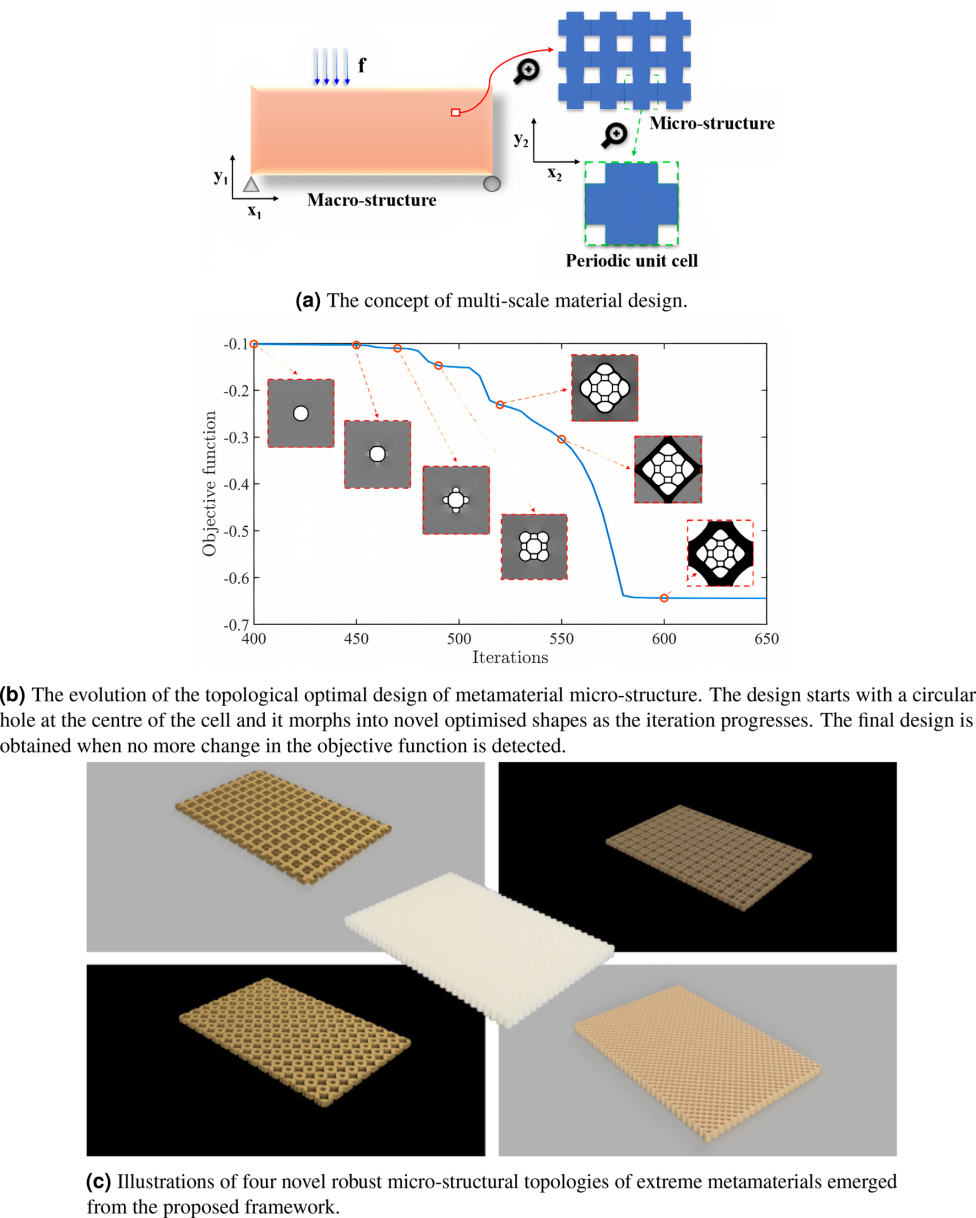
An illustrative visual tool guide of the key aspects of the proposed design paradigm. The focus is on optimizing the topology of the periodic unit cell as shown in (**a**) and determine robust micro-structural designs against uncertainties. Note that the objective of the deterministic topological design in (**b**) is to maximize the bulk modulus (refer Table 1 for details). The negative ordinate indicates the maximization problem. The 3D computer aided design (CAD) geometries of the robust micro-structural configurations shown in (**c**) are obtained using Autodesk Fusion 360 software (version 2.0.9930) (https://www.autodesk.co.uk/campaigns/education/fusion-360). These geometries can be directly exported for 3D printing. The four different geometries shown here correspond to robust topologies exhibiting extreme mechanical properties (for example, maximum bulk modulus, maximum shear modulus, minimum Poisson’s ratio and maximum equivalent elastic modulus).

For exploring such exciting possibilities of discovering new materials with extreme properties, determining their optimal configuration at the very conceptual micro-structural design stage is indispensable^7^. A recent works with multi-material lattices^8^, pre-stressed materials^9^ and piezo-embedded damped lattice metamaterials^10,11^ show some ways of achieving extreme homogeneous properties. In this scenario, topology optimization (TO) is well-known to be an effective computational analysis tool which renders greater design freedom and yields the best material layout given a prescribed design domain and boundary condition^12^. Depending on the algorithm’s performance and versatility, various successful TO methods have emerged to the present^13^, few popular ones being the solid isotropic material with penalization (SIMP), evolutionary structural optimization (ESO), level set method (LSM), and moving morphable components (MMC). In fact, the popularity of TO has even led to its integration with relatively newer numerical discretization schemes (compared to finite element method) such as, isogeometric analysis^14^. A simple illustration of multi-scale design and topology optimization of the micro-structure of metamaterial is presented in Fig. 1a and b, respectively.

The success of TO is clearly evident from its extensive applications for material micro-structural design in recent years. In this regard, few note-worthy literatures have been discussed as follows. Gibiansky and Sigmund^15^ investigated multi-phased elastic composites and obtained topological design corresponding to extreme bulk and shear modulus. For obtaining maximum bulk and shear modulus, the optimal topology of cellular micro-structures was determined using ESO^16^. A compact code in MATLAB based on the SIMP and energy-based homogenization method (EBHM) was developed for extreme material multi-scale structural design^17^. Clausen et al.^18^ designed and fabricated 3D auxetic material micro-structures undergoing large deformations. Long et al.^19^ performed topology optimization to maximize the effective Young’s modulus, so as to obtain the optimal distribution of 3D material micro-structures whose constituent phases consist of non-identical Poisson’s ratios. A novel TO method was presented based on the independent point-wise density interpolation to obtain a bi-material chiral metamaterial^20^. Chen and Huang^21^ designed 3D chiral metamaterials based on the couple-stress homogenization and maximized the chiralty (the coupling effects between tension/compression and torsion) of the cellular structure. Xu et al.^14^ achieved designs of ultra-lightweight architected materials along with extremal properties such as, maximum bulk and shear modulus by using isogeometric TO. Petal-shaped auxetic metamaterials were designed where a back-propagation neural network was coupled with isogeometric TO to evaluate their macroscopic equivalent properties^22^. Zheng et al.^23^ proposed two numerical schemes within the evolutionary optimization framework to obtain 2D auxetic metamaterials with favourable characteristics, and experimentally validated their special properties. Ye et al.^24^ proposed a method for the simultaneous adaptive design of gradually stiffer mechanical metamaterials along with auxetic property.

Although the above published articles related to micro-structural TO explore multiple potent research prospects to achieve extreme material properties, they are restricted to the deterministic optimization framework. In this regard, the literature is found to be scarce in studies addressing uncertainties in manufacturing processes. The following few works highlight the importance of considering manufacturing variability and their effect on the structural design. Most nano, micro and macro structures fabricated using e-beam lithography, etching and milling processes, respectively are vulnerable to manufacturing uncertainties. Even the structures meticulously designed and optimized may experience inferior performance or in an extreme scenario, lose their functionality due to the wear of machining tools, under or over-etching, or malcalibrated e-beam equipment. To address few of the above aspects, robust topology optimization (RTO) for structural design was presented accounting for manufacturing errors in^25–27^. Specifically, the effect of over-etching (erosion) and under-etching (dilation) was modelled for structures produced by milling or etching, which may cause the structural parts to become thinner or thicker than intended. For the works^25,26^, the problem was posed as a worst case design formulation and the error effects were simulated by a Heaviside projection threshold. While they considered only uniform manufacturing errors (i.e. constant in magnitude over the entire design domain), the method was extended to account for non-uniform manufacturing errors (i.e. with spatially varying magnitude)^27^. For doing so, a probabilistic approach was followed where the projection threshold was represented by a (non-Gaussian) random field. Jansen et al.^28^ developed an RTO framework which incorporated the spatially varying geometric imperfections due to misalignment and misplacement of material often encountered in slender (civil engineering) structures. These imperfections should be accounted for in accordance to the provision recommended in the Eurocode for design of steel structures and by the Joint Committee on Structural Safety. Note that the misplacement errors cause a perturbation in the spatial location of the material, whereas the errors due to etching, add or remove material at the structural surface. An ESO based RTO framework was developed for multi-material structures considering interval loading uncertainty in^29^. Efficiency was enhanced by (1) orthogonal decomposition of the loading uncertainty which decoupled the stochastic problem from the optimization loop and (2) sensitivity analysis. Material uncertainty modelled as imprecise probability was integrated with the multi-scale concurrent ESO based TO framework in^30^. The RTO methodology adopted the type I hybrid random interval model. An ESO based RTO framework was developed for multiscale 2D and 3D structures considering multiple random loading conditions in^31^. Decoupled sensitivity analysis was observed to improve the computational efficiency. An ESO based RTO methodology was proposed for actuator-coupled structures considering hybrid uncertainties in^32^. The hybrid random interval model simulated the stochastic mechanical and piezoelectric parameters and perturbation analysis was employed to evaluate the statistical quantities.

While the above works simulated manufacturing uncertainties numerically and investigated their effect on the structural topology, the following studies have carried out experimental analysis to probe manufacturing uncertainties. It has been shown that the emission rate of a 3D printer based on fused deposition modeling principle increased significantly with the increase in the extrusion temperature^33^. Melenka et al.^34^ found that the per cent infill has a significant effect on the longitudinal elastic modulus and ultimate strength of the test specimens, whereas print orientation and layer thickness fail to achieve significance. Dimensional analysis of test specimens also showed that the test specimen varied significantly from the nominal print dimensions. The manufacturing tolerances of metamaterial samples produced by a selective laser sintering process were obtained to simulate the manufacturing uncertainty in mass-produced industrial applications^35^. It was found that even small levels of variability, given by less than 1 % for the mass and less than 3 % for the elastic modulus, has a significant effect on the overall vibration attenuation performance.

While the first of the above three paragraphs discusses works on deterministic TO for extreme metamaterial design, the second paragraph illustrates the numerical simulation of manufacturing uncertainty and robust topological design. Finally, the above paragraph highlights a few instances of experimental exploration of manufacturing uncertainties. Motivated by the need to consider manufacturing uncertainties for metamaterial design, this work attempts to bridge the above points. Thus, we integrate these aspects to present an RTO formulation specifically for extreme metamaterial design by minimizing the effect of potential material uncertainties. To the best of the authors’ knowledge, this is one of the first applications of robust micro-structural design for obtaining extreme mechanical properties of metamaterials. Few instances of ready-to-print robust micro-structural topologies of extreme metamaterials achieved as a part of this study have been presented in Fig. 1c for illustrative purpose and the interest of readers.

## Methodology

**Figure 2:**
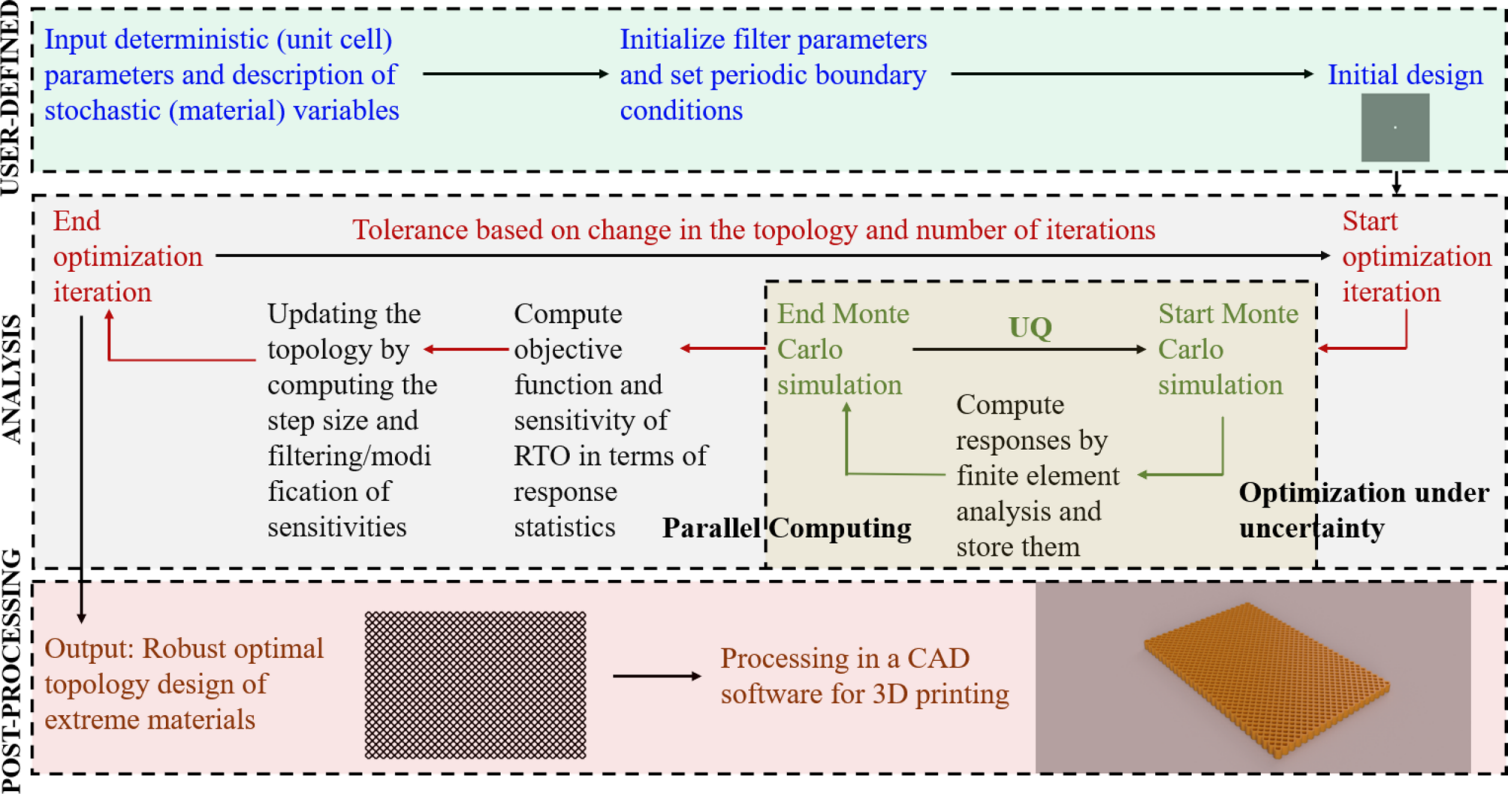
Flowchart of the proposed framework. The three modules, namely, (1) user-defined initialization, (2) topology optimization under uncertainty analysis and (3) post-processing unit for additive manufacturing, are bordered by dotted lines which constitute the approach. A square-shaped void region at the centre of the 150 $\times$ 150 unit cell is adopted as the initial design (as shown in the right side of the user-defined module block). The robust optimal topology obtained (corresponding to maximum shear modulus subjected to the spatial variability of the elastic modulus (refer Table 2)) is shown as a 20 $\times$ 30 periodic arrangement of the unit cell in the post-processing module. The 3D fabrication-ready object is obtained after extruding and material rendering in Autodesk Fusion 360 software (version 2.0.9930) (https://www.autodesk.co.uk/campaigns/education/fusion-360). The abbreviations RTO and UQ represent robust topology optimization and uncertainty quantification, respectively.

The work integrates the material uncertainty model (simulated by in-house developed codes) with deterministic topology optimization^36^ to develop a parallel Monte Carlo based robust topology optimization framework. A flow-diagram of the proposed RTO framework is presented in Fig. 2. The main intent is to illustrate the effect of material uncertainties on the micro-structural topologies and derive robust configurations for extreme material design. As shown in Fig. 2, the entire methodology comprises of three steps: (1) the user-defined input module where the system and the optimization parameters are initialized, (2) the analysis module where the expensive computations take place involving uncertainty quantification nested within the optimization loop. To reduce the computational cost, a parallel version of Monte Carlo simulation is employed to simulate the randomness within each optimization iteration. The details of this step regarding the optimization algorithm, finite element model, energy-based homogenization and sensitivity computations are presented subsequently. (3) The post-processing module is where the resulting robust micro-structural topologies with extreme properties are exported to a CAD software for 3D printing.

### Homogenization

Considering linear elastic behaviour, the equivalent constitutive behaviour of periodic structures can be determined by homogenization technique. This work employs the energy-based homogenization approach to predict the macroscopic equivalent elastic properties of micro-structures^36^. The approach is based upon energy conservation with respect to stress and strain. The homogenized stiffness tensor $E^{H}_{ijkl}$ can be expressed in terms of mutual energies as

1 $$\begin{aligned} E^{H}_{ijkl} = \frac{1}{|Y|}\int _{Y}E_{pqrs}\varepsilon _{pq}^{A(ij)}\varepsilon _{rs}^{A(kl)}dY \end{aligned}$$

where *Y* represents the base cell, $E_{pqrs}$ is the elasticity tensor in index notation, $\varepsilon _{pq}^{A(ij)}$ and $\varepsilon _{rs}^{A(kl)}$ denote the superimposed strain fields. When the base cell is discretized into *N* finite elements, Eq. (1) can be approximated as

2 $$\begin{aligned} E^{H}_{ijkl} = \frac{1}{|Y|}\sum _{e=1}^{N} (\varvec{u}_{e}^{A(ij)})^{T}\mathbf {k}_{e}\varvec{u}_{e}^{A(kl)} \end{aligned}$$

where $\varvec{u}_{e}^{A(kl)}$ are the element displacement solutions corresponding to the unit test strain fields $\varepsilon ^{0(kl)}$ and $\mathbf {k}_{e}$ denotes the element stiffness matrix. It is to be noted that for 2-D problems, $11 \equiv 1$, $22 \equiv 2$, and $12 \equiv 3$. Thus, Eq. (2) can be concisely represented as

3 $$\begin{aligned} Q_{ij} = \frac{1}{|Y|}\sum _{e=1}^{N} q_{e}^{(ij)} \end{aligned}$$

where $Q_{ij}$ is given by

4 $$\begin{aligned} \begin{bmatrix} Q_{11} &{} Q_{12} &{} Q_{13}\\ Q_{21} &{} Q_{22} &{} Q_{23}\\ Q_{31} &{} Q_{32} &{} Q_{33} \end{bmatrix} = \begin{bmatrix} E_{1111}^{H} &{} E_{1122}^{H} &{} E_{1112}^{H}\\ E_{2211}^{H} &{} E_{2222}^{H} &{} E_{2212}^{H}\\ E_{1211}^{H} &{} E_{1222}^{H} &{} E_{1212}^{H} \end{bmatrix} \end{aligned}$$

In Eq. (3), $q_{e}^{(ij)}$ are the element mutual energies and can be expressed as

5 $$\begin{aligned} q_{e}^{(ij)} = (\varvec{u}_{e}^{A(i)})^{T}\mathbf {k}_{e}\varvec{u}_{e}^{A(j)} \end{aligned}$$

### Periodic boundary conditions

The displacements on a pair of opposite boundaries of a 2-D base cell

6 $$\begin{aligned} \begin{aligned} u_{i}^{k-}&= \varepsilon _{ij}^{0}y_{j}^{k-} + u_{i}^{*}\\ u_{i}^{k+}&= \varepsilon _{ij}^{0}y_{j}^{k+} + u_{i}^{*} \end{aligned} \end{aligned}$$

where notations $k-$ and $k+$ represent the pair of two opposite parallel boundary surfaces perpendicular to the $k{{\rm th}}$ direction. One can remove the unknown periodic fluctuation term $u_{i}^{*}$ by using the difference between the displacements as,

7 $$\begin{aligned} \begin{aligned} u_{i}^{k+} - u_{i}^{k-} = \varepsilon _{ij}^{0}(y_{j}^{k+} - y_{j}^{k-}) \end{aligned} \end{aligned}$$

For a given unit cell, the quantity $(y_{j}^{k+} - y_{j}^{k-})$ is constant. Thus, the right side of Eq. (7) is a constant for a specified $\varepsilon _{ij}^{0}$. This boundary conditions can be incorporated within the finite element model by restraining the corresponding pairs of nodal displacements. Note that this boundary configuration satisfies the periodicity and continuity conditions in terms of displacement and stress employing displacement-based finite element analysis^37^.

### Optimization approach

The unit cell is discretized into *N* finite elements, resulting into equal number of density design variables $\rho \in \mathbb {R}^{N}$. The element level elastic modulus can be defined by the modified SIMP method^38^ as

8 $$\begin{aligned} \begin{aligned} {E_e(\rho _{e}) = E_{\text {min}} + \rho _{e}^{p}(E_{0}-E_{\text {min}})} \end{aligned} \end{aligned}$$

where $E_{\text {min}}$ denotes the elastic modulus of the Ersatz material, an approximation for void material to prevent singularity of the stiffness matrix, $0<\rho _{e}<1$, limits corresponding to Ersatz and solid materials, *p* is a penalization factor for driving the density towards the black and white solution and $E_0$ denotes the elastic modulus of solid material.

The deterministic optimization problem can be expressed as

9 $$\begin{aligned} \begin{aligned} \underset{ \rho }{\text {minimize}}\;&c(E_{ijkl}^{H}(\rho ))\\ \text {subject to}\;&{\mathbf {K}{\varvec{u}}^{A(kl)} = {\varvec{f}}^{kl}}, \; k, l = 1, \ldots , d \\&\sum _{e=1}^{N} v_{e}\rho _{e}/|Y|\le v, \; 0\le \rho _{e}\le 1, \; e = 1, \ldots , N. \end{aligned} \end{aligned}$$

where the objective function $c(E_{ijkl}^{H})$ is a function of the homogenized stiffness tensors, $\mathbf {K}$ is the global stiffness matrix, $\varvec{u}^{A(kl)}$ and $\varvec{f}^{kl}$ are the global displacement and external force vectors of the test case (*kl*), respectively, *d*, $v_e$ and *v* denote the spatial dimension, element volume and upper limit of the volume fraction, respectively.

The motive of this work is to determine the extreme properties of material micro-structures, such as maximum bulk modulus, maximum shear modulus, minimum Poisson’s ratio and maximum equivalent elastic modulus. The corresponding objective functions can be defined as

- Design of maximum bulk modulus:
  10 $$\begin{aligned} c = -(E_{1111} + E_{1122} + E_{2211} + E_{2222}) \end{aligned}$$
- Design of maximum shear modulus:
  11 $$\begin{aligned} c = -E_{1212} \end{aligned}$$
- Design of minimum Poisson’s ratio:
  12 $$\begin{aligned} c = E_{1122} + E_{2211} -\delta E_{1212} \end{aligned}$$
- Design of maximum equivalent elastic modulus:
  13 $$\begin{aligned} c = -\frac{1}{2}(E_{1111} + E_{2222}) \end{aligned}$$

Note that the topological design of minimum and negative Poisson’s ratio $(E_{1122}/E_{1111})$ has always been a difficult issue. It is observed from the literature that auxetic properties can be obtained by imposing additional constraints on isotropy or bulk modulus^36^. An equivalent expression in the form of Eq. (12) is used for this purpose and adopted from^23^. Selection of the term $\delta$ in Eq. (12) is discussed later in the numerical illustration section.

The solution scheme for solving the equilibrium equation in Eq. (9) adopted here can be found in Xia and Breitkopf^36^. After obtaining the finite element displacement solution, the optimization problem in Eq. (9) is solved by using optimality criteria method. The heuristic updating criterion^13^ is expressed as

14 $$\begin{aligned} \begin{aligned} \rho _{e}^{\text {new}} = \;&\text {max}(0,\rho _{e}-m), \; \text {if} \; \rho _{e}B_{e}^{\eta } \le \text {max}(0,\rho _{e}-m) \\ \;&\text {min}(0,\rho _{e}+m), \; \text {if} \; \rho _{e}B_{e}^{\eta } \ge \text {min}(0,\rho _{e}+m) \\ \;&\rho _{e}B_{e}^{\eta }, \; \text {otherwise} \end{aligned} \end{aligned}$$

where *m* is the step size, $\eta$ is the damping coefficient and $B_e$ can be determined by using the following optimality condition

15 $$\begin{aligned} \begin{aligned} B_{e} = \frac{-\frac{\partial c}{\partial \rho _{e}}}{\lambda \frac{\partial V}{\partial \rho _{e}}} \end{aligned} \end{aligned}$$

where $\lambda$ is the Lagrange multiplier. It imposes the satisfaction of the material volume fraction constraint. The numerator $\frac{\partial c}{\partial \rho _{e}}$ which is the sensitivity of the objective function can be determined by using the adjoint method as

16 $$\begin{aligned} \begin{aligned} \frac{\partial E_{ijkl}^{H}}{\partial \rho _{e}} = \frac{1}{|Y|}p\rho _{e}^{p-1}(E_{0}-E_{\text {min}})(\varvec{u}_{e}^{A(ij)})^{T}\mathbf {k}_{0}\varvec{u}_{e}^{A(kl)} \end{aligned} \end{aligned}$$

where $\mathbf {k}_{0}$ represent the element stiffness matrix with unit elastic modulus. For a uniform mesh, the element volume $v_e = 1$ and hence, $\frac{\partial V}{\partial \rho _{e}} = 1$. To ensure convergence of the optimization problem Eq. (9), filtering techniques are used which are illustrated next.

### Filtering

To eliminate the formation of a checkerboard pattern and resolve the mesh-dependent issue, a filter is applied either to the sensitivities or the densities^38,39^. The sensitivity based filtering modifies the sensitivities $\frac{\partial c}{\partial \rho _{e}}$ (Eq. (16) as,

17 $$\begin{aligned} \frac{\widehat{\partial c}}{\partial \rho _{e}} = \frac{1}{\text {max}(\gamma ,\rho _{e})\sum _{i \in N_e} H_{ei}} \sum _{i \in N_e} H_{ei}\rho _{i}\frac{\partial c}{\partial \rho _{i}} \end{aligned}$$

where $\gamma = 10^{-3}$ is a small positive number incorporated to avoid the denominator becoming zero. $H_{ei}$ is a weight term defined in Eq. (18). $N_e$ represents the set of elements *i* whose centre-to-centre distance to element *e*, $\Delta (e,i)$, is less than the filter radius $r_{\text {min}}$. The size of the filter radius can be used to control the minimum size of the emerging features in the design domain.

18 $$\begin{aligned} H_{ei} = \text {max}(0,r_{\text {min}}-\Delta (e,i)) \end{aligned}$$

The density based filtering converts the original densities to the filtered densities $\rho _e$ as,

19 $$\begin{aligned} \tilde{\rho }_e = \frac{1}{\sum _{i \in N_e} H_{ei}} \sum _{i \in N_e} H_{ei}\rho _{i} \end{aligned}$$

The sensitivities with respect to $\rho _j$ can be obtained as,

20 $$\begin{aligned} \frac{\partial c}{\partial \rho _j} = \sum _{e \in N_j} \frac{\partial c}{\partial \tilde{\rho }_e} \frac{\partial \tilde{\rho }_e}{\partial \rho _j}=\sum _{e \in N_j} \frac{1}{\sum _{i \in N_e} H_{ei}} H_{ej} \frac{\partial c}{\partial \tilde{\rho }_{e}} \end{aligned}$$

Note that $\frac{\partial c}{\partial \tilde{\rho }_{e}}$ can be evaluated by Eq. (16) upon replacing $\rho _e$ with $\tilde{\rho }_e$.

### Robust topology optimization

The objective function considered for the robust topology optimization is

21 $$\begin{aligned} c_{\text {RTO}} = \mu _c + 3\sigma _c \end{aligned}$$

where $\mu _c$ and $\sigma _c$ are the mean and standard deviation of the deterministic objective function *c*, respectively.

### Sensitivity calculation

Differentiating $c_{\text {RTO}}$, we obtain

22 $$\begin{aligned} \frac{\partial c_{\text {RTO}}}{\partial d_e} = \frac{\partial \mu _c}{\partial d_e} + 3\frac{\partial \sigma _c}{\partial d_e}. \end{aligned}$$

Now considering the first term of Eq. (22) and expanding it, we obtain

23 $$\begin{aligned} \begin{aligned} \frac{\partial \mu _c}{\partial d_e}&= \frac{\partial }{\partial d_e} \left[ \frac{1}{N_{\text {simu}}}\sum _{i=1}^{N_{\text {simu}}}{c_i} \right] = \frac{1}{N_{\text {simu}}}\sum _{i=1}^{N_{\text {simu}}}{\frac{\partial c_i}{\partial d_e}} = \mathbb {E}\left( \frac{\partial c}{\partial d_e} \right) \end{aligned} \end{aligned}$$

where we have represented the expectation based on $N_{\text {simu}}$ number of samples. From Eq. (23), we can see that the first term of Eq. (22) can be computed based on the expectation of the topological derivatives in the deterministic case. For the second term in Eq. (22), we have

24 $$\begin{aligned} \begin{aligned} \frac{\partial \sigma _c}{\partial d_e} = \frac{\partial \sqrt{{\mathop {\mathrm{var}}} \left( c\right) }}{\partial d_e}&= \frac{1}{2\sqrt{{\mathop {\mathrm{var}}} \left( c\right) }} \frac{\partial }{\partial d_e}\left[ \mathop {\mathrm{var}}\left( c\right) \right] = \frac{1}{2\sqrt{{\mathop {\mathrm{var}}} \left( c\right) }} \frac{\partial }{\partial d_e}\left[ \mathbb {E}\left( c^2\right) - \mathbb {E}\left( c\right) ^2\right] \\&= \frac{1}{2\sqrt{{\mathop {\mathrm{var}}} \left( c\right) }} \left\{ \left[ \frac{\partial }{\partial d_e}\mathbb {E}\left( c^2\right) \right] - \left[ \frac{\partial }{\partial d_e}\mathbb {E}\left( c\right) ^2\right] \right\} \\ \end{aligned} \end{aligned}$$

where $\mathop {\mathrm{var}}\left( \bullet \right)$ indicates the *variance* operator. Considering the first term in Eq. (24) and expanding it similar to Eq. (23), we have

25 $$\begin{aligned} \begin{aligned} \frac{\partial }{\partial d_e}\mathbb {E}\left( c^2\right) =&\frac{\partial }{\partial d_e} \left[ \frac{1}{N_{\text {simu}}}\sum _{i=1}^{N_{\text {simu}}}{c_i^2}\right] = \frac{1}{N_{\text {simu}}}\sum _{i=1}^{N_{\text {simu}}}{\frac{\partial c_i^2}{\partial d_e}} = \frac{1}{N_{\text {simu}}}\sum _{i=1}^{N_{\text {simu}}}{2c_i\frac{\partial c_i}{\partial d_e}} = 2\mathbb {E}\left( c\frac{\partial c}{\partial d_e} \right) . \end{aligned} \end{aligned}$$

Finally, considering the second term in Eq. (24) and using Eq. (23), we have

26 $$\begin{aligned} \begin{aligned} \frac{\partial }{\partial d_e}\mathbb {E}\left( c\right) ^2&= 2\mathbb {E}\left( c\right) \frac{\partial }{\partial d_e}\mathbb {E}\left( c\right) = 2\mathbb {E}\left( c\right) \mathbb {E}\left( \frac{\partial c}{\partial d_e} \right) . \end{aligned} \end{aligned}$$

Substituting Eqs. (26) and (25) into Eq. (24), we obtain

27 $$\begin{aligned} \frac{\partial \sigma _c}{\partial d_e} = \frac{1}{\sqrt{{\mathop {\mathrm{var}}} \left( c\right) }} \left[ \mathbb {E}\left( c\frac{\partial c}{\partial d_e} \right) - \mathbb {E}\left( c\right) \mathbb {E}\left( \frac{\partial c}{\partial d_e} \right) \right] . \end{aligned}$$

Finally, substituting Eqs. (27) and (23) into Eq. (22), we obtain

28 $$\begin{aligned} \frac{\partial c_{\text {RTO}}}{\partial d_e} = \mathbb {E}\left( \frac{\partial c}{\partial d_e} \right) + 3\left[ \frac{1}{\sqrt{{\mathop {\mathrm{var}}} \left( c\right) }} \left\{ \mathbb {E}\left( c\frac{\partial c}{\partial d_e} \right) - \mathbb {E}\left( c\right) \mathbb {E}\left( \frac{\partial c}{\partial d_e} \right) \right\} \right] . \end{aligned}$$

From Eq. (28), it is obvious that the topological derivatives for RTO can be computed based on conventional/deterministic topological derivatives.

## Numerical illustration: results and discussion

### Implementation details

In this work, the design domain is considered to be square and discretized into square plane stress elements. The volume fraction is taken to be 0.5. The unit cell is discretized into $3 \times 3$ elements. The influence of an initial design can affect the final optimal topology significantly. For this work, a square shaped void region at the center of the unit cell is adopted as the initial design^40^ as shown in Fig. 2. The small void is generally inserted in the initial design to stimulate the evolutionary optimization process.

It has been reported in multiple studies that topology optimization for the design of extreme material properties is prone to multiple local minima. The final optimal solution is found to be extremely sensitive to the initial topology, shape of the unit cell, filter radius $(r_{\text {min}})$, penalization factor (*p*), filtering approach (sensitivity filtering or density filtering) and others. Therefore, for selecting the appropriate parameter values, few rules of thumb have been followed. The optimization problem Eq. (9) is non-convex for $p\ge 1$. Although the high values of penalization factor will result in more distinct topologies, the algorithm is likely to get trapped in a local minima^17^. A smaller filter radius value generally results in a better solution (in terms of a detailed micro-structure) as higher frequency details are visible through low-pass filter. Compared to sensitivity filtering, density filtering is observed to yield less sensitive designs and hence preferable for material micro-structure design.

Parametric study has been carried out by considering various combinations of stochastic material parameter models such as, spatial variation via random field and random parametric models. The stochastic models assumed are in line with the literature (discussed in the introduction section) where spatially varying and parametric models simulate non-homogenous and uniform manufacturing errors, respectively. For stochastic simulations, the elastic modulus and Poisson’s ratio are considered to be random and follow log-normal distribution with 5$\%$ variability. It is practical to assume that all realizations corresponding to these random parameters will be positive and therefore, they are assumed to be log-normally distributed. In doing so, 1000 MCS is performed within every optimization iteration to simulate the material variability. This has been carried out in a cost-effective manner using parallel computing (employing all 8 cores of an Intel Xeon processor). Four case studies have been undertaken to obtain extreme properties of material micro-structures, such as maximum bulk modulus, maximum shear modulus, minimum negative Poisson’s ratio (auxetic metamaterials) and maximum equivalent elastic modulus.

The initial design is the same for all the following cases studied (except type III). The nominal values of the elastic modulus and Poisson’s ratio at the element level are adopted as 1 and 0.3, respectively. For the stochastic cases, their mean values are same as that of the nominal values. The length scale used for discretizing the random field model is taken as 0.2. The results are represented by reporting the optimal unit cell, $3 \times 4$ periodic arrangement, equivalent elastic matrices and robust optimal solutions for each of the extreme material configurations.

### Maximum bulk modulus (Type I)

Table 1 illustrates the optimal solutions for the case of type I. To obtain these results, density based filtering and the following parameter values are considered: $p=5$ and, $r_{\text {min}}=2$. It can be observed from Table 1 that uncertainty affects both the topological configuration and the equivalent elastic matrix. The stiffness terms $Q_{11}$ and $Q_{22}$ are observed to increase for all types of uncertainty models compared to the deterministic case. Specifically, the greatest rise is observed for the random field cases. The term $Q_{33}$ decreases due to uncertainty. The off-diagonal term $Q_{12}$ reduces for all uncertainty models and the deterministic model achieves the maximum value. Interestingly, for the hybrid input uncertainty modelling case, the resulting structure shows non-zero (negative) $Q_{13}$ and $Q_{23}$ terms. This indicates coupling of axial and shear deformations/stress states. Although there is a slight difference between the terms $Q_{11}$ and $Q_{22}$ for the deterministic and random field models, the difference is significant for the hybrid uncertainty model, exhibiting strong anisotropic behaviour. In terms of mean value of the objective function, the random field cases lead to higher value of bulk modulus compared to the deterministic and parametric uncertainty cases. Also, the random field cases result in a more robust topological design indicated by lower values of the standard deviation of objective function.

**Table 1:**
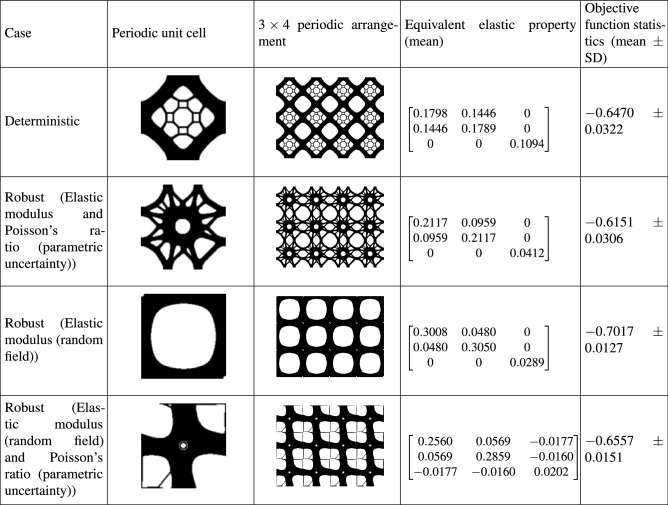
Optimal solutions for maximum bulk modulus (Type I).

Results from four case studies are shown. They include, (1) deterministic design, (2) robust design considering parametric uncertainty models for elastic modulus and Poisson’s ratio, (3) robust design considering random field model for elastic modulus and (4) robust design considering a combination of random field and parametric uncertainty models for the elastic modulus and Poisson’s ratio, respectively. Note that the terms of the equivalent elastic matrix (given by Eq. (4)) represent their mean values. The standard deviation of the matrix terms is consistent with the level of input uncertainty and is observed to be within 5% bounds. The objective functions of the deterministic and robust optimization have been evaluated by Eqs. (10) and (21), respectively.

### Maximum shear modulus (Type II)

Table 2 illustrates the optimal solutions for the case of type II. To obtain these results, density based filtering and the following parameter values are considered: $p=5$ and, $r_{\text {min}}=2$.

**Table 2:**
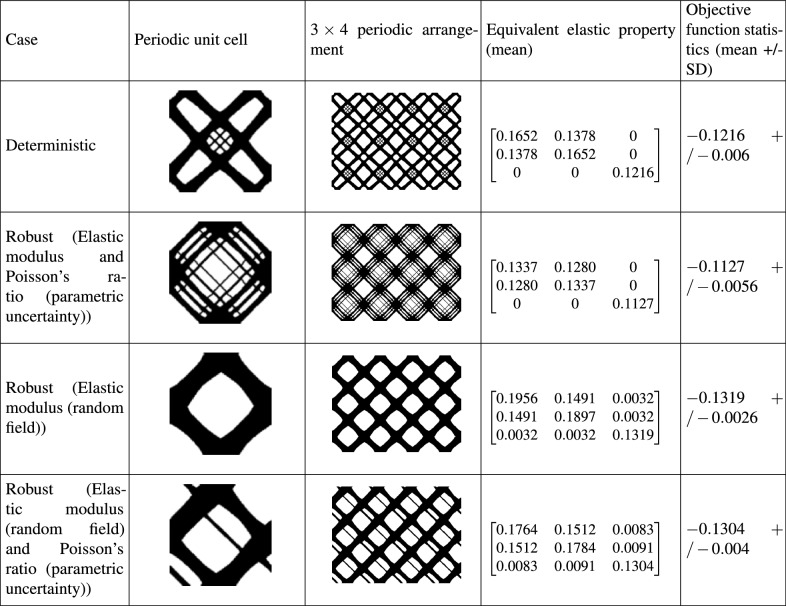
Optimal solutions for maximum shear modulus (Type II).

Results from four case studies are shown. They include, (1) deterministic design, (2) robust design considering parametric uncertainty models for elastic modulus and Poisson’s ratio, (3) robust design considering random field model for elastic modulus and (4) robust design considering a combination of random field and parametric uncertainty models for the elastic modulus and Poisson’s ratio, respectively. Note that the terms of the equivalent elastic matrix (given by Eq. (4)) represent their mean values. The standard deviation of the matrix terms is consistent with the level of input uncertainty and is observed to be within 5 % bounds. The objective functions of the deterministic and robust optimization have been evaluated by Eqs. (11) and (21), respectively.

It can be observed from Table 2 that uncertainty affects both the topological configuration and the equivalent elastic matrix. The stiffness terms (diagonal entries) $Q_{11}$, $Q_{22}$ and $Q_{33}$ and the off-diagonal term $Q_{12}$ are observed to increase for the random field and hybrid input uncertainty models compared to the deterministic case. These terms achieve lower values for the parametric uncertainty model compared to the deterministic case. Interestingly, for the random field and hybrid input uncertainty models, the resulting structure show (1) non-zero values of $Q_{13}$ and $Q_{23}$ terms and (2) $Q_{11} \ne Q_{22}$. (1) indicates coupling of axial and shear deformations/stress states and (2) illustrates anisotropic behaviour. In terms of mean value of the objective function, the random field and hybrid input uncertainty models lead to higher value of shear modulus compared to the deterministic and parametric uncertainty cases. The parametric stochastic model results in the minimum value of shear modulus. Also, the random field and hybrid input uncertainty models result in a more robust topological design indicated by lower values of the standard deviation of objective function.

### Minimum Poisson’s ratio (Type III)

Density based filtering and the following parameter values are considered: $p=5$ and, $r_{\text {min}}=5$, for the type III case. The unit cell is discretized into 200 elements along both the horizontal and vertical directions. As the term $\delta$ (refer Eq. (12)) causes significant sensitivity to the topological design of auxetic metamaterials^23^, a parametric study is performed in Table 3 to investigate the effect of $\delta$ on the resulting topologies with minimum Poisson’s ratio.

**Table 3:** Parametric study to determine $\delta$ in Eq. (12) to achieve minimum negative Poisson’s ratio (Type III).

$\delta$	Response statistics	Deterministic	Robust (parametric uncertainty)	Robust (random field)	Robust (hybrid uncertainty)
0.5	Mean of objective function	− 0.0392	− 0.0336	− 0.0950	− 0.1024
	SD of objective function	0.0019	0.0017	0.0019	0.0019
	Mean of Poisson’s ratio	− 0.4915	− 0.4791	− 0.5895	− 0.5908
1.0	Mean of objective function	− 0.0405	− 0.0355	− 0.1098	− 0.1110
	SD of objective function	0.0020	0.0018	0.0025	0.0025
	Mean of Poisson’s ratio	− 0.4168	− 0.3293	− 0.5468	− 0.5518
1.5	Mean of objective function	− 0.0559	− 0.0508	− 0.1260	− 0.1249
	SD of objective function	0.0028	0.0025	0.0028	0.0029
	Mean of Poisson’s ratio	0.0965	0.0831	− 0.6120	− 0.5376
2.0	Mean of objective function	− 0.0912	− 0.0828	− 0.1304	− 0.1336
	SD of objective function	0.0045	0.0041	0.0030	0.0031
	Mean of Poisson’s ratio	0.1423	0.1553	− 0.5269	− 0.5318
2.5	Mean of objective function	− 0.1190	− 0.1142	− 0.1274	− 0.1260
	SD of objective function	0.0059	0.0065	0.0029	0.0029
	Mean of Poisson’s ratio	0.1552	0.1769	− 0.3299	− 0.3872

Results from four case studies are shown. They include, (1) deterministic design (column 3), (2) robust design considering parametric uncertainty models for elastic modulus and Poisson’s ratio (column 4), (3) robust design considering random field model for elastic modulus (column 5) and (4) robust design considering a hybrid combination of random field and parametric uncertainty models for the elastic modulus and Poisson’s ratio (column 6). Note that the mean Poisson’s ratio is computed by $(E_{1122}/E_{1111})$ from the mean equivalent elastic matrix. The objective functions of the deterministic and robust optimization have been evaluated by Eqs. (12) and (21), respectively.

It can be observed from Table 3 that out of the adopted $\delta$ values, only $\delta$ = 0.5 and 1 lead to negative mean Poisson’s ratio corresponding to the deterministic and each of the stochastic models. $\delta$ = 0.5 has been selected as lower Poisson’s ratios are obtained compared to the case $\delta$ = 1. The corresponding row has been highlighted to indicate the best and the most consistent performance in Table 3 for clarity. Table 4 illustrates the optimal solutions for the case of type III, corresponding to $\delta$ = 0.5.

**Table 4:**
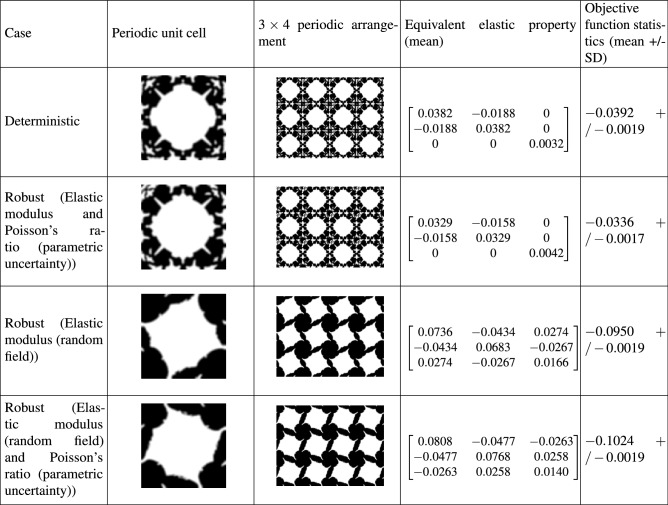
Optimal solutions for minimum Poisson’s ratio (Type III).

Results from four case studies are shown. They include, (1) deterministic design, (2) robust design considering parametric uncertainty models for elastic modulus and Poisson’s ratio, (3) robust design considering random field model for elastic modulus and (4) robust design considering a combination of random field and parametric uncertainty models for the elastic modulus and Poisson’s ratio. Note that the terms of the equivalent elastic matrix (given by Eq. (4)) represent their mean values. The standard deviation of the matrix terms is consistent with the level of input uncertainty and is observed to be within 5 % bounds. The objective functions of the deterministic and robust optimization have been evaluated by Eqs. (12) and (21), respectively.

It can be observed from Table 4 that uncertainty affects both the topological configuration and the equivalent elastic matrix. Although the overall resulting topologies from the deterministic and parametric uncertainty models are very similar with fine differences at the boundary. The stiffness terms $Q_{11}$ and $Q_{22}$ are observed to increase for the random field and hybrid uncertainty models and decrease for parametric uncertainty compared to the deterministic case. The stiffness term $Q_{33}$ is observed to increase for all stochastic models compared to the deterministic case. The off-diagonal term $Q_{12}$ is negative for all the cases reported and observed to increase for the parametric stochastic model and decrease for the random field and hybrid uncertainty cases than that of the deterministic one. Interestingly, for the random field and hybrid input uncertainty models: (1) the terms $Q_{13}$ and $Q_{23}$ are non-zero (negative), which illustrates coupling of axial and shear deformations/stress states and (2) $Q_{11} \ne Q_{22}$, which indicates anisotropic behaviour. The random field and hybrid input uncertainty models lead to lower values of the mean objective function compared to the deterministic and parametric uncertainty cases. Specifically, the parametric and hybrid stochastic models result in the maximum and minimum value of Poisson’s ratio, respectively. The deterministic, random field and hybrid input uncertainty models result in the same level of robustness of the topological design indicated by the standard deviation of the objective function. The parametric uncertainty model achieves the most robust design by a small margin. The results in Table 4 illustrate that negative Poisson’s ratio (given by $Q_{12}/Q_{11}$) has been achieved for all the case studies undertaken. This is worth mentioning as the design of auxetic metamaterials by topology optimization is a challenging task on its own and even more difficult with uncertainties.

### Maximum equivalent elastic modulus (Type IV)

Table 5 illustrates the optimal solutions for the case of type IV. To obtain these results, density based filtering and the following parameter values are considered: $p=5$ and, $r_{\text {min}}=2$.

**Table 5:**
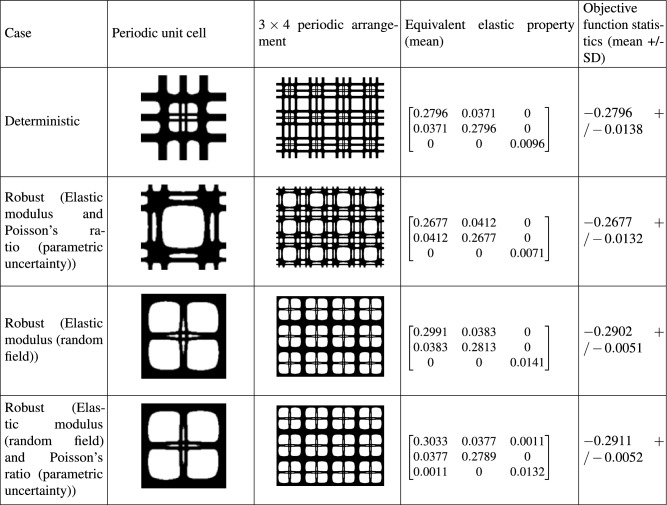
Optimal solutions for maximum equivalent elastic modulus (Type IV). Results from four case studies are shown.

They include, (1) deterministic design, (2) robust design considering parametric uncertainty models for elastic modulus and Poisson’s ratio, (3) robust design considering random field model for elastic modulus and (4) robust design considering a combination of random field and parametric uncertainty models for the elastic modulus and Poisson’s ratio, respectively. Note that the terms of the equivalent elastic matrix (given by Eq. (4)) represent their mean values. The standard deviation of the matrix terms is consistent with the level of input uncertainty and is observed to be within 5 % bounds. The objective functions of the deterministic and robust optimization have been evaluated by Eqs. (13) and (21), respectively.

It can be observed from Table 5 that uncertainty affects both the topological configuration and the equivalent elastic matrix. The resulting topologies obtained from the random field and hybrid random input models are the same. The stiffness terms (diagonal entries) $Q_{11}$, $Q_{22}$ and $Q_{33}$ are observed to increase for the random field compared to the deterministic case. The stiffness terms $Q_{11}$ and $Q_{33}$ are observed to increase for the hybrid random input model compared to the deterministic case. These terms achieve lower values for the parametric uncertainty model compared to the deterministic case. The off-diagonal term $Q_{12}$ is observed to be higher for all the stochastic models than that of the deterministic one. Interestingly, for the hybrid input uncertainty models, the resulting structure shows anisotropic property indicated by $Q_{11} \ne Q_{22}$ and illustrates coupling of axial and shear deformations/stress states indicated by non-zero $Q_{13}$ term. In terms of mean value of the objective function, the random field and hybrid input uncertainty models lead to higher value of equivalent elastic modulus compared to the deterministic and parametric uncertainty cases. The parametric stochastic model results in the minimum value of equivalent elastic modulus. Also, the random field and hybrid input uncertainty models result in a more robust topological design indicated by lower values of the standard deviation of objective function.

### Conclusions

This section summarizes the results obtained from the numerical investigation. Few critical points have been enumerated below:

- It has been observed that the material uncertainties affect the resulting micro-structural topologies and mechanical performance significantly.
- Out of the input stochastic material models, the random field model followed by the hybrid one lead to desirable performance in terms of (1) optimal mean performance, (2) most robust performance and (3) elastic property, indicated by the mean value of objective function, standard deviation of objective function and stiffness terms of the equivalent elastic matrix, respectively, for types I, II and IV.
- For type III, the hybrid uncertainty model followed by the random field leads to desirable output performance (based upon combination of the above points (1)–(3)). This point along with the above are indicative of the degree of sensitivity of input stochastic models on the optimal micro-structural topology. Thus, these observations have a practical relevance as one needs to be more cautious in fabricating the metastructures which follow a more sensitive input stochastic model.
- The robust design leads to improved material properties compared to the deterministic design.
- Multiple interesting topological patterns have been found for guiding the robust metamaterial design. Importantly, all of the resulting topologies have tessellating property, which make them feasible for 3D printing. Thus, there can be a possible extension of the present computational work as the resulting robust topological configuration of materials with extreme properties are practically realizable.
- In addition to the few resulting micro-structural configurations showing isotropic properties, interestingly, the others yielded configurations which showed anisotropic property. Some cases led to positive and negative coupling of axial and shear deformations/stress states. These phenomenon were primarily observed for the random field and hybrid uncertainty cases. This observation may be attributed to the fact that the spatial variability (inhomogeneity) in the micro-structure induces anisotropy and coupling phenomena in the material. Although this requires further investigation and achieving this was unintentional but will prove to be useful for designing material micro-structures with dual/simultaneous properties.
- Considering the fact that the design of auxetic metamaterials by topology optimization is a challenging task on its own and even becomes more difficult to obtain robust designs with micro-structural uncertainties, it is worth noting that negative Poisson’s ratio was achieved for all the undertaken cases of type III using the proposed framework by adopting appropriate parameters (Table 4). However, one should be careful in choosing the parameters considering their high sensitivity on the resulting topologies (for example, $\delta$ of Eq. (12)) as evident from Table 3. It is also recommended to thoroughly scrutinize the existing equivalent expressions for minimizing the Poisson’s ratio and is highly sensitive to the problem type or application in hand.
- The effect of uncertainties on the material micro-structures is so high that even considering the accuracy and high precision of additive manufacturing techniques, accounting nominal amount of uncertainty for material design is recommended, at least in the conceptual design stage before the manufacturing. In fact, there is nothing at stake, as it is shown in this study that robustness improves the design in terms of mechanical performance.

## Data Availability

All relevant data are available from the corresponding author upon reasonable request, and/or are included within the main part and Supplementary Information.
